# Enhanced Transmissibility and Decreased Virulence of HIV-1 CRF07_BC May Explain Its Rapid Expansion in China

**DOI:** 10.1128/spectrum.00146-22

**Published:** 2022-06-21

**Authors:** Zetao Cheng, Huanchang Yan, Qingmei Li, Sherimay D. Ablan, Alex Kleinpeter, Eric O. Freed, Hao Wu, Emmanuel Enoch Dzakah, Jianhui Zhao, Zhigang Han, Haiying Wang, Shixing Tang

**Affiliations:** a Guangdong Provincial Key Laboratory of Tropical Disease Research, School of Public Health, Southern Medical University, Guangzhou, China; b Guangzhou Center for Disease Control and Prevention, Guangzhou, China; c Virus-Cell Interaction Section, HIV Dynamics and Replication Program, Center for Cancer Research, National Cancer Institute, Frederick, Maryland, USA; d Institute of Public Health, Guangzhou Medical University & Guangzhou Center for Disease Control and Prevention, Guangzhou, China; Thomas Jefferson Univeristy

**Keywords:** HIV-1 genotype, CRF07_BC, transmission cluster, p6 mutation, virus release, infectivity, replication, Alix protein

## Abstract

HIV-1 CRF07_BC is one of the most common circulating recombinant forms (CRFs) in China and is becoming increasingly prevalent especially in HIV-infected men who have sex with men (MSM). The reason why this strain expanded so quickly in China remains to be defined. We previously observed that individuals infected with HIV-1 CRF07_BC showed slower disease progression than those infected with HIV-1 subtype B or CRF01_AE. CRF07_BC viruses carry two unique mutations in the p6Gag protein: insertion of PTAPPE sequences downstream of the original Tsg101 binding domain, and deletion of a seven-amino-acid sequence (30PIDKELY36) that partially overlaps with the Alix binding domain. In this study, we confirmed the enhanced transmission capability of CRF07_BC over HIV-1 subtype B or CRF01_AE by constructing HIV-1 transmission networks to quantitatively evaluate the growth rate of transmission clusters of different HIV-1 genotypes. We further determined lower virus infectivity and slower replication of CRF07_BC with aforementioned PTAPPE insertion (insPTAP) and/or PIDKELY deletion (Δ7) in the p6Gag protein, which in turn may increase the pool of people infected with CRF07_BC and the risk of HIV-1 transmission. These new features of CRF07_BC may explain its quick spread and will help adjust prevention strategy of HIV-1 epidemic.

**IMPORTANCE** HIV-1 CRF07_BC is one of the most common circulating recombinant forms (CRFs) in China. The question is why and how CRF07_BC expanded so rapidly remains unknown. To address the question, we explored the transmission capability of CRF07_BC by constructing HIV-1 transmission networks to quantitatively evaluate the growth rate of transmission clusters of different HIV-1 genotypes. We further characterized the role of two unique mutations in CRF07_BC, PTAPPE insertion (insPTAP) and/or PIDKELY deletion (Δ7) in the p6Gag in virus replication. Our results help define the molecular mechanism regarding the association between the unique mutations and the slower disease progression of CRF07_BC as well as the quick spread of CRF07_BC in China.

## INTRODUCTION

Since the first report of the confirmed human immunodeficiency virus type 1 (HIV-1) infection in 1985 in China, HIV-1 transmission modes have dramatically changed from original blood transmission including drug abuse to sexual contact, in particular homosexual transmission among men who have sex with men (MSM) (1). The prevalence of HIV-1 infection in MSM has rapidly increased from 0.9% in 2003 to 7.8% in 2016. In 2017, 25.5% of the 34358 newly diagnosed HIV-1 infections were from MSM (1, 2). In addition, the composition of HIV-1 genotypes has been changing significantly and is dominated by CRF01_AE and CRF07_BC as well as the emerging CRF55_01B in China (3). CRF07_BC, a circulating recombinant form (CRF) of HIV-1, was first identified in 2000 and has become one of the most commonly transmitted strains among MSM in China (4–6). From 2006 to 2012, the prevalence of CRF07_BC increased from 12.5% to 43.2% in Shenzhen (7). Another study in Jiangsu province indicated that the proportion of CRF07_BC infections rapidly increased from 13.0% to 39.4% between 2012 and 2015 (8). However, an unanswered question is why and how CRF07_BC expanded so quickly in China?

We and others have reported that CRF07_BC infection exhibited lower viral loads and slower disease progression compared with individuals infected with HIV-1 subtype B or CRF01_AE. Our results also confirmed the differential rates of immunodeficiency progression as a function of HIV-1 genotypes including subtype B, CRF01_AE and CRF07_BC (2). We found that CRF07_BC infected patients exhibited lower risk of disease progression to immunodeficiency, i.e. CD4 cell count < 200 cells/ml and lower immunodeficiency incidence compared to those infected with HIV-1 subtype B or CRF01_AE while the period from HIV-1 diagnosis to the occurrence of immunodeficiency was significantly longer for CRF07_BC than CRF55_01B and CRF01_AE (2, 9–11). These findings suggested that CRF07_BC infection may slow disease progression, which in turn may increase the number of people living with HIV-1 and increase the transmission probability of CRF07_BC infection. In 2007, Lin et al. described a unique deletion mutation of 7 amino acids (aa) (30PIDKELY36) in the p6^Gag^ protein of HIV-1 CRF07_BC isolates (12). This 7-aa sequence partially overlaps with the host ALG-2 interacting protein X (Alix) binding domain and may affect virus production (12). In the previous study, we downloaded in LANL HIV-1 database a total of 296 CRF07_BC sequences collected from 1997 to 2013 and found that 54% of CRF07_BC sequences carry this signature Δ7 mutation(p6Δ7), and intriguingly, the prevalence of the p6Δ7 variant increased over time from 23.5% during 1997–2002, 46.0% during 2003–2007 to 61.1% during 2008–2013 (13). In addition, we found that 26% of CRF07_BC p6Δ7 variant viruses carry another insertion mutation of 6-aa, PTAPPE (insPTAP), in the p6Gag protein downstream of the PT/SAP motif, which serves as the binding site for the host protein tumor susceptibility gene 101 (Tsg101)(13). Of note, this mutation sequence was first reported in 2005 (GenBank no. KU050275) when the p6Δ7 variant was becoming the dominant variant among CRF07_BC virus. However, the role of these two mutations of Δ7 and insPTAP in determining HIV-1 transmission and in regulating HIV-1 replication remains to be characterized.

In this study, we explored the transmission capability of CRF07_BC by constructing HIV-1 transmission networks to quantitatively evaluate the growth rate of transmission clusters of different HIV-1 genotypes. We further characterized CRF07_BC with aforementioned PTAPPE insertion (insPTAP) and/or PIDKELY deletion (Δ7) in the p6^Gag^ protein. Our results help define the molecular mechanism regarding the association between the unique mutations and the slower disease progression of CRF07_BC as well as the quick spread of CRF07_BC in China.

## RESULTS

### Sequences of CRF07_BC form the largest HIV-1 transmission cluster with high growth rate.

**FIG 1:**
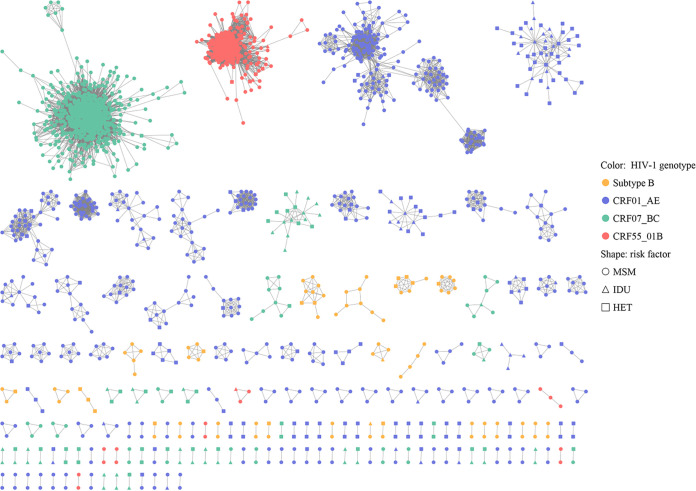
The HIV-1 network in Guangzhou, China.

A total of 2,786 HIV-1 *pol* sequences were collected from HIV-infected subjects of four most prevalent HIV-1 genotypes in Guangzhou including subtype B (174, 5.65%), CRF01_AE (1093, 35.50%), CRF07_BC (1155, 37.48%), and CRF55_01B (364, 11.82%) and were used for constructing HIV-1 transmission clusters. Using a threshold of pairwise genetic distance of 1.5%, 74.26% (2069/2786) of HIV-1 sequences clustered with at least one other sequence to form 172 HIV-1 transmission clusters (Fig. 1). The size of these clusters ranged from 2 to 784. Among them, 97 clusters had only two members (Fig. 1; Fig. S1 in the supplemental material). The three largest clusters were Cluster 1 (CRF07_BC, *n* = 784), Cluster 126 (CRF55_01B, *n* = 336), and Cluster 2 (CRF01_AE, *n* = 190), respectively.

The cluster growth rate values ranged from 0 to 8.42 with a median value of 1.57 (IQR 0.00-5.28) and presented as a right-skewed distribution (Fig. S2 in the supplemental material). These clusters were further divided into high (*n* = 664), medium (*n* = 633), and low growth clusters (*n* = 1,328) with a cluster growth rate of ≥5.28, 1.57–5.28, and <1.57, respectively. They were significantly different with regard to gender, age, marital status, education level, occupation, infection routes and HIV-1 genotypes (Table 1; *P* < 0.001). Of note, 98.04% of the subjects in the high growth clusters infected with CRF07_BC while none of the individuals infected with HIV-1 subtype B or CRF01_AE were found in the rapid growth clusters. In contrast, 12.05% and 60.77% of the subjects infected with HIV-1 subtype B or CRF01_AE were in the low growth clusters, respectively (Table 1). Furthermore, the routes of HIV-1 infection were significantly different among the three classes of HIV-1 growth clusters. The proportion of the subjects infected through homosexual transmission was 56.85%, 84.31%, and 92.32% in the low, medium, and high growth clusters, respectively (*P* < 0.001, Table 1).

**TABLE 1:** Characteristics of subjects among different HIV-1 transmission growth clusters^*a*^

Characteristics	Growth rate of HIV-1 transmission cluster (*n*, %)				*P* value
	Low (*n* = 1,328)	Medium (*n* = 663)	High (*n* = 664)	Total (*n* = 2,655)	
Gender					<0.001
Male	1,178 (88.70)	645 (97.29)	662 (99.70)	2,485 (93.60)	
Female	150 (11.30)	18 (2.71)	2 (0.30)	170 (6.40)	
Age group					<0.001
≤20	52 (3.92)	30 (4.52)	48 (7.23)	130 (4.90)	
21~30	568 (42.77)	309 (46.61)	348 (52.41)	1,225 (46.14)	
31~40	384 (28.92)	215 (32.43)	186 (28.01)	785 (29.57)	
41~	324 (24.4)	109 (16.44)	82 (12.35)	515 (19.40)	
Marital status					<0.001
Unmarried	772 (58.13)	412 (62.14)	483 (72.74)	1,667 (62.79)	
Married	405 (30.80)	174 (26.24)	136 (20.48)	719 (27.08)	
Divorced	83 (6.25)	40 (6.03)	35 (5.27)	158 (5.95)	
Data missing	64 (4.82)	37 (5.58)	10 (1.51)	111 (4.18)	
Education level					<0.001
Primary or below	171 (12.88)	38 (5.73)	30 (4.52)	239 (9.00)	
Junior or high	685 (51.58)	319 (48.11)	321 (48.34)	1,325 (49.91)	
College or others	416 (31.33)	272 (41.03)	307 (46.23)	995 (37.48)	
Data missing	56 (4.22)	34 (5.13)	6 (0.90)	96 (3.62)	
Occupation					<0.001
Unskilled work	603 (45.41)	332 (50.08)	331 (49.85)	1,266 (47.68)	
Skilled/Professional work	85 (6.40)	69 (10.41)	65 (9.79)	219 (8.25)	
Student	53 (3.99)	34 (5.13)	46 (6.93)	133 (5.01)	
Unemployment	276 (20.78)	75 (11.31)	63 (9.49)	414 (15.59)	
Not disclosed	311 (23.42)	153 (23.08)	159 (23.95)	615 (23.47)	
Infection routes					<0.001
HET	334 (25.15)	53 (12.97)	41 (6.18)	461 (17.36)	
PWID	239 (18.00)	18 (2.71)	10 (1.51)	267 (10.06)	
MSM	755 (56.85)	559 (84.31)	613 (92.32)	1927 (72.58)	
HIV-1 genotype				<0.001	
Subtype B	160 (12.05)	4 (0.60)	0 (0.00)	164 (6.18)	
CRF01_AE	870 (60.77)	232 (34.99)	0 (0.00)	1,039 (39.13)	
CRF07_BC	328 (24.70)	116 (17.50)	650 (98.04)	1,094 (41.21)	
CRF55_01B	33 (2.48)	311 (46.91)	13 (1.96)	358 (13.48)	

a Cluster growth at diagnosis was categorized as high (≥75th percentile), medium (>50th to <75th percentile), and low (≤50th percentile). HET, heterosexually transmitted; MSM, men who have sex with men; PWID, people who inject drugs.

### CRF07_BC is associated with the quick transmission of HIV-1.

Further regression analysis showed that HIV-infected subjects from medium and high growth clusters (growth rate ≥ 1.57) were more likely to be infected with CRF07_BC (OR = 14.11; 95% CI, 11.16–17.85; *P* < 0.001) or CRF55_01B (OR = 41.17; 95% CI, 26.94–62.91; *P* < 0.001). Due to the absence of individuals infected with subtype B and CRF01_AE in the high growth clusters, further analysis was restricted to individuals infected by CRF07_BC and CRF55_01B with the outcome of fast cluster growth. The results indicated that CRF07_BC-infiected individuals were more likely located in the fast growth clusters whereas HIV-infected individuals with high growth (growth rate ≥ 5.28) were less likely to be infected by HIV-1 CRF55_01B (OR = 0.014; 95% CI, 0.008–0.025; *P* < 0.001; Table 2). Stepwise analysis also confirmed HIV-1 genotype, in particular CRF07_BC, as the main factor associated with fast growth of HIV-1 clusters (*P* < 0.001, Table 3).

**TABLE 2:** Factors associated with the growth index of HIV-1 transmission clusters using overall models

Characteristics	Cluster growth ≥ 1.57		Cluster growth ≥ 5.28	
	OR (95% CI)	*P*	OR (95% CI)	*P*
Gender				
Male	ref		ref	
Female	0.267 (0.146, 0.490)	<0.001	0.072 (0.016, 0.319)	<0.001
Age group				
Below 20	1.279 (0.771, 2.121)	0.341	1.323 (0.675, 2.591)	0.323
21~30	ref		ref	
31~40	0.906 (0.687, 1.193)	0.482	0.956 (0.655, 1.394)	0.779
41~	0.565 (0.393, 0.811)	0.002	0.747 (0.445, 1.256)	0.186
Marital status				
Unmarried	ref		ref	
Married	1.132 (0.824, 1.555)	0.445	1.239 (0.793, 1.937)	0.199
Divorced	1.315 (0.795, 2.174)	0.286	1.537 (0.756, 3.126)	0.251
Not disclosed	1.167 (0.614, 2.219)	0.638	0.612 (0.210, 1.780)	0.540
Education level				
Primary or below	ref		ref	
Junior or high	1.018 (0.655, 1.582)	0.935	1.020 (0.550, 1.893)	0.728
College or others	1.137 (0.697, 1.855)	0.607	1.056 (0.539, 2.068)	0.975
Not disclosed	1.292 (0.590, 2.831)	0.522	0.228 (0.062, 0.842)	0.612
Occupation				
Unskilled work	ref		ref	
Skilled/professional work	1.630 (1.086, 2.448)	0.018	1.200 (0.688, 2.092)	0.157
Students	1.060 (0.637, 1.765)	0.822	1.018 (0.513, 2.019)	0.436
Unemployment	0.899 (0.633, 1.275)	0.549	1.014 (0.629, 1.634)	0.141
Not disclosed	1.024 (0.776, 1.352)	0.867	1.225 (0.828, 1.813)	0.936
Infection routes				
MSM	ref		ref	
HET	0.323 (0.226, 0.461)	<0.001	0.192 (0.119, 0.310)	<0.001
PWID	0.044 (0.027, 0.072)	<0.001	0.025 (0.012, 0.052)	<0.001
Genotypes				
B	0.074 (0.027, 0.202)	<0.001		
CRF01_AE	ref			
CRF07_BC	14.113 (11.160, 17.847)	<0.001	ref	
CRF55_01B	41.170 (26.942, 62.911)	<0.001	0.014 (0.008, 0.024)	<0.001

**TABLE 3:** Factors associated with the growth index of HIV-1 transmission clusters using stepwise models^*a*^

Characteristics	Cluster growth ≥ 1.57		Cluster growth ≥ 5.28	
	OR (95% CI)	*P*	OR (95% CI)	*P*
Gender				
Male	ref		ref	
Female	0.277 (0.152, 0.503)	<0.001	0.083 (0.019, 0.361)	0.001
Age group				
Below 20	1.223 (0.743, 2.012)	0.428		
21~30	ref			
31~40	0.942 (0.729, 1.216)	0.644		
41~	0.600 (0.436, 0.826)	0.002		
Education level				
Primary or below			ref	
Junior or high			1.103 (0.600, 2.026)	0.753
College or others			1.145 (0.605, 2.168)	0.677
Not disclosed			0.178 (0.057, 0.552)	0.003
Occupation				
Unskilled work	ref			
Skilled/professional work	1.664 (1.122, 2.470)	0.011		
Students	1.104 (0.675, 1.807)	0.693		
Unemployment	0.896 (0.632, 1.270)	0.538		
Not disclosed	1.051 (0.804, 1.374)	0.717		
Infection routes				
MSM	ref		ref	
HET	0.329 (0.234, 0.463)	<0.001	0.188 (0.121, 0.294)	<0.001
PWID	0.043 (0.027, 0.069)	<0.001	0.023 (0.012, 0.046)	<0.001
Genotypes				
B	0.074 (0.027, 0.203)	<0.001		
CRF01_AE	ref			
CRF07_BC	14.015 (11.092, 17.710)	<0.001	ref	
CRF55_01B	41.220 (27.002, 62.925)	<0.001	0.014 (0.008, 0.025)	<0.001

a The factors were removed from the multivariate model if *P* > 0.1, using stepwise progress. HET, heterosexually transmitted; MSM, men who have sex with men; PWID, people who inject drugs.

To strengthen the robustness of the analysis, sensitivity analysis restricted to HIV-infected MSM also confirmed the association between CRF07_BC infection and quick growth of HIV-1 transmission clusters (*P* < 0.001; Table S1 in the supplemental material). Stepwise analysis further indicated the significant association between HIV-1 cluster growth and HIV-1 genotype (*P* < 0.001; Table S2). The ranking order of HIV-1 genotypes in affecting the growth of HIV-1 transmission clusters was: CRF07_BC > CRF55_01B > CRF01_AE > subtype B (Table 4; *P* < 0.001), which was consistent with the paired comparison results obtained in MSM (Table S3).

**TABLE 4:** Comparison of HIV-1 transmission growth index according to HIV-1 genotypes^*a*^

HIV-1 genotype	HIV-1 transmission growth index			*H*, *P* value*
	No. analyzed (%)	Median (quartile)	Mean rank	
Subtype B	164 (6.18)	0.00 (0.00, 0.25)	659.95	*H* = 869.70, *P* < 0.001
CRF01_AE	1,039 (39.13)	0.00 (0.00, 1.34)	896.87	
CRF07_BC	1,094 (41.21)	5.58 (0.00, 6.03)	1,747.48	
CRF55_01B	358 (13.48)	3.41 (2.83, 4.28)	1603.41	
Total	2655 (100.00)	1.57 (0.00, 5.28)	1328	

a Cluster growth was measured by calculating the number of newly diagnosed cases linked in a cluster in the previous 12 months divided by the square root of the cluster size. * Kruskal-Wallis test (post hoc test by Bonferroni).

### HIV-1 CRF07_BC with p6^Gag^ mutation inhibited virus particle release.

**FIG 2:**
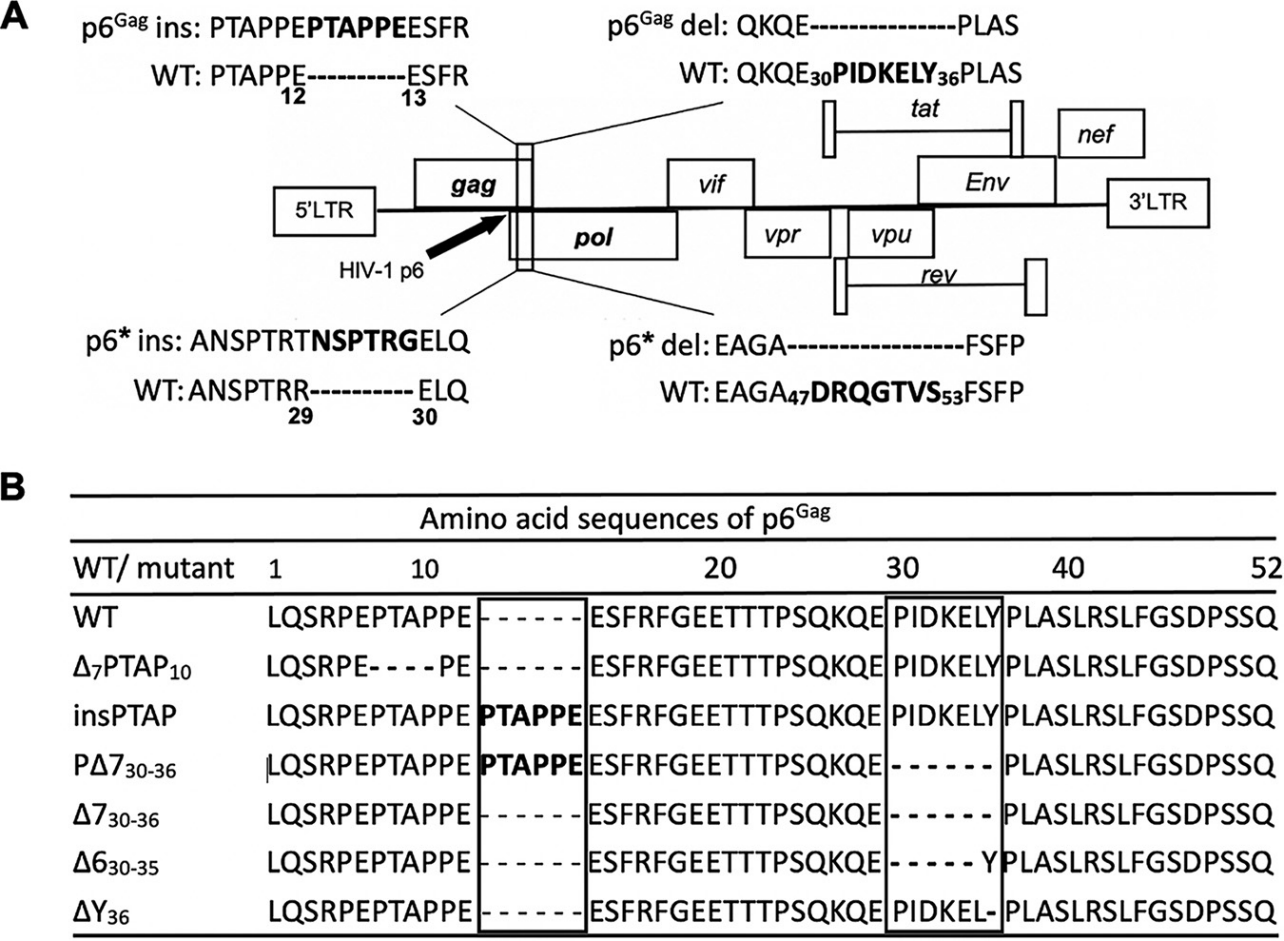
Schematic of the HIV-1 genome and p6 mutants. (A) The HIV-1 genome is shown with an expanded view of p6 mutations including PTAPPE insertion and PIDKELY deletion in p6^Gag^, as well as NSPTRG insertion and DRQGTVS deletions in p6*. (B) HIV-1 wild-type(WT) and p6^Gag^ mutants. ΔPTAP, a mutant bearing the deletion of the original _7_PTAP_10_ motif was constructed and used as a control for deficiency of virus release; insPTAP, PTAPPE insertion; Δ7, _30_PIDKELY_36_ deletion in p6^Gag^; PΔ7, the double mutation bearing insPTAP and Δ7; ΔY, deletion of the 36th residue in p6^Gag^; and Δ6, _30_PIDKEL_35_ deletion in p6^Gag^. Sequences of mutations are highlighted and compared with HIV-1 WT. To help distinguish the mutants, we included the first and last amino acid numbers for some mutants in this figure, but not shown in other figures.

**FIG 3:**
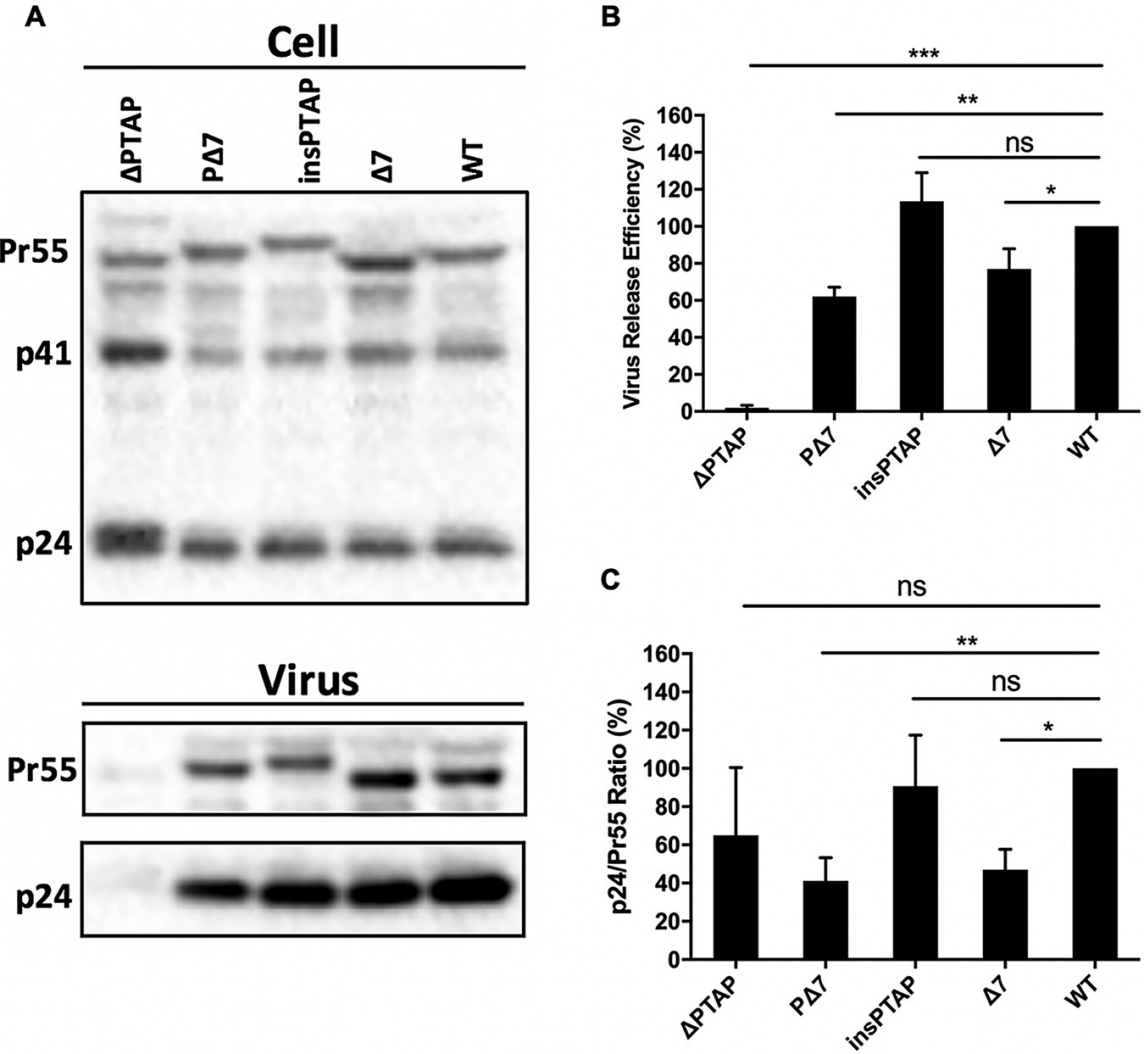
Virus release efficiency and Gag processing. (A) Western blotting (WB) analysis. 293T cells were transfected with WT pNL4-3 or p6^Gag^ mutants. At 24 h post-transfection, cell and virus lysates were collected and analyzed by WB with HIV immunoglobulin (HIV-Ig). Positions of HIV-1 Gag precursor Pr55^Gag^, Gag processing intermediate p41 and HIV-1 capsid protein p24 are indicated. (B) Virus release efficiency. The relative efficiency of virus release was calculated as the amount of virion p24 divided by total Gag (virion p24 + cellular Pr55Gag + cellular p24). (C) Gag processing. Gag processing was expressed as the ratio of p24 relative to Pr55^Gag^ in virions. The data were plotted in bar graphs. The efficiency of virus release and Gag processing for WT was set as 100%. Error bars indicate the standard deviation (SD) from more than three independent experiments; ns, not significant. *, *P* < 0.05, **, *P* < 0.01, and ***, *P* < 0.001.

Because we hypothesized that slow disease progression caused by decreased virus replication and infectivity may be associated with enlarged pool and enhanced transmission of CRF07_BC in particular the virus with the signature mutations at p6^Gag^ protein. Therefore, we assessed the virological characteristics of different HIV-1 clones by introducing mutations insPTAP and Δ7, as well as the double mutation PΔ7 p6^Gag^ mutations into the full-length HIV-1 molecular clone (Fig. 2). We also created a deletion mutation of the Tsg101-binding motif PTAP (ΔPTAP) as a control of defect in HIV-1 assembly and release. As expected, ΔPTAP mutation severely impaired virus particle production (Fig. 3B) but did not impact Gag cleavage in cell (Fig. 3A, upper panel) and virion (Fig. 3C). The insPTAP mutation downstream of the original PTAP motif did not significantly affect virus release (Fig. 3B). The Δ7 and the double mutation PΔ7 moderately inhibited virus production to ~77% and ~62% of the WT level, respectively (Table 5, Fig. 3B). Similar results were obtained when the reverse transcriptase (RT) activity of the culture supernatants was measured to determine the efficiency of virus release (Table 5).

**TABLE 5:** Phenotypes of HIV-1 wild-type and p6 mutant virions

Virus^*a*^	Virus release^*b*^		Maturation (p24/Pr55) %	Infectivity^*b*^ RLU^*e*^ (%)	Replication kinetics^*f*^ days of peak (% RT level)^*g*^			
	RT^*c*^ (%)	VRE^*d*^ ^(^%)			Sup-T1	MT-4	PBMC-D1	PBMC-D2
WT	100	100	100	100	11 (100.0)	4 (100.0)	7 (100)	9 (100)
ΔPTAP	15.9 ± 14.6	1.9 ± 1.4	65.0 ± 35.5		17 (31.3)	7 (29.4)	Defective	Defective
PΔ7	63.9 ± 26.1	62.1 ± 5.1	41.1 ± 12.1	69.1 ± 18.6	28 (14.8)	6 (64.1)	7 (39.9)	9 (16.0)
insPTAP	108.8 ± 25.3	113.4 ± 15.4	90.8 ± 26.7	113.5 ± 20.9	13 (116.8)	4 (132.4)	7 (96.7)	9 (85.0)
Δ7	73.0 ± 11.4	76.9 ± 11.0	47.0 ± 10.6	56.3 ± 3.6	21 (34.6)	6 (64.7)	7 (59.0)	10 (29.1)

a WT, wild type; ΔPTAP, deletion of original PTAP motif; insPTAP, insertion of PTAPPE sequences; Δ7, deletion of 7-aa in p6; PΔ7, double mutation of insPTAP and Δ7.

b The levels of p6 mutants were expressed as a percentage relative to WT level, which was arbitrarily set as 100%. Data were shown as mean value with standard deviation (SD).

c RT, reverse transcriptase activity. Data are shown as mean ± SD.

d VRE, virus release efficiency, is calculated as the amount of viron-p24 (CA) divided by the total amount of Gag (see Materials and Methods). Data are shown as mean ± SD.

e RLU, relative luciferase unit.

f Replication Kinetics, replication capacity of each mutant in T-cell lines (Sup-T1 and MT-4) or T-cells (PBMC Donor 1 and 2).

g % RT level, the peak level of each mutant was normalized to WT, which is set as 100. All the data presented in Table 5 are summarized from Figure 3 and Figure 4.

### HIV-1 CRF07_BC with p6^Gag^ mutation decreased virus replication and infectivity.

We further investigated the infectivity of these p6^Gag^ mutants and found that the insPTAP mutant showed similar levels of virus infectivity to HIV-1 WT in the TZM-bl system (Table 5). The infectivity of the mutant Δ7 and the double mutant PΔ7 was ~56% and 69% WT level, respectively (Table 5). Furthermore, the ΔPTAP mutant showed very low-level and delayed replication kinetics in the T-cell lines (Fig. 4A and B), and no replication was observed in PBMCs (Fig. 4C and D). The insPTAP mutant was replication competent in both T-cell lines and PBMCs with no major difference from HIV-1 WT (Fig. 4). However, the Δ7 and PΔ7 mutants were replication impaired in virus replication in SupT1 and MT-4 T cells (Fig. 4A and B, Table 5), while low-level replication was also observed in PBMCs from two different donors (Fig. 4C and D, Table 5).

**FIG 4:**
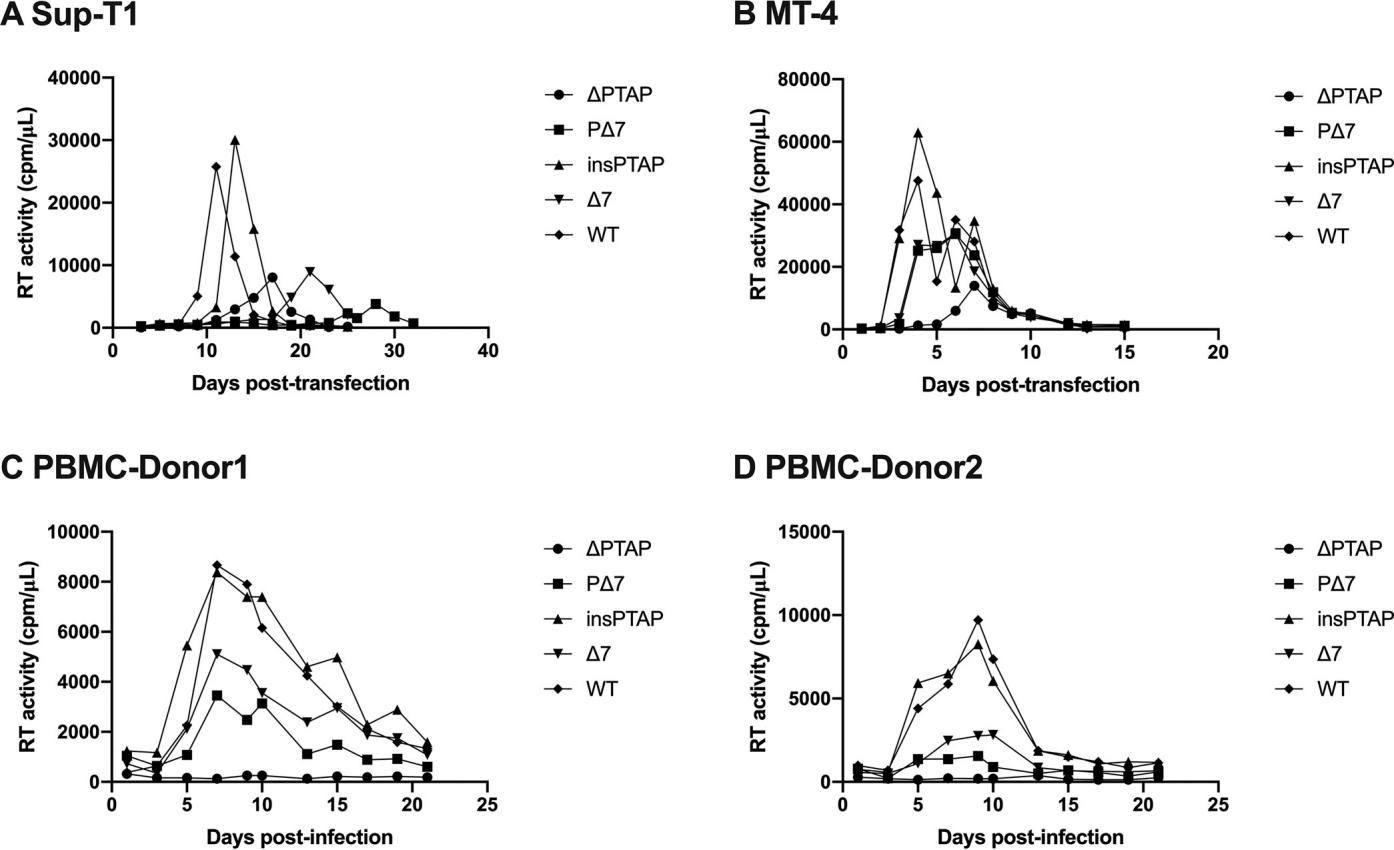
Replication of HIV-1 WT and p6 mutants in Sup-T1 T cells (A), MT-4 T cells (B), and PBMCs (C and D). Both Sup-T1 and MT-4 T-cell lines were transfected with WT or mutant pNL4-3 molecular clones. Cells were split every day or every two days. Culture supernatants were collected for reverse transcriptase (RT) activity analysis. PBMCs from two donors were infected with virus stocks generated in 293T cells. Cells were split, and culture supernatants collected for RT activity analysis every 2-3 days. Virus replication was monitored by RT activity. Data in SupT1 and MT4 cell lines were obtained from two independent experiments and data in PBMCs cells were obtained only from one experiment.

### The defects of HIV-1 CRF07_BC were caused by p6^Gag^ mutation, not p6* protein.

**FIG 5:**
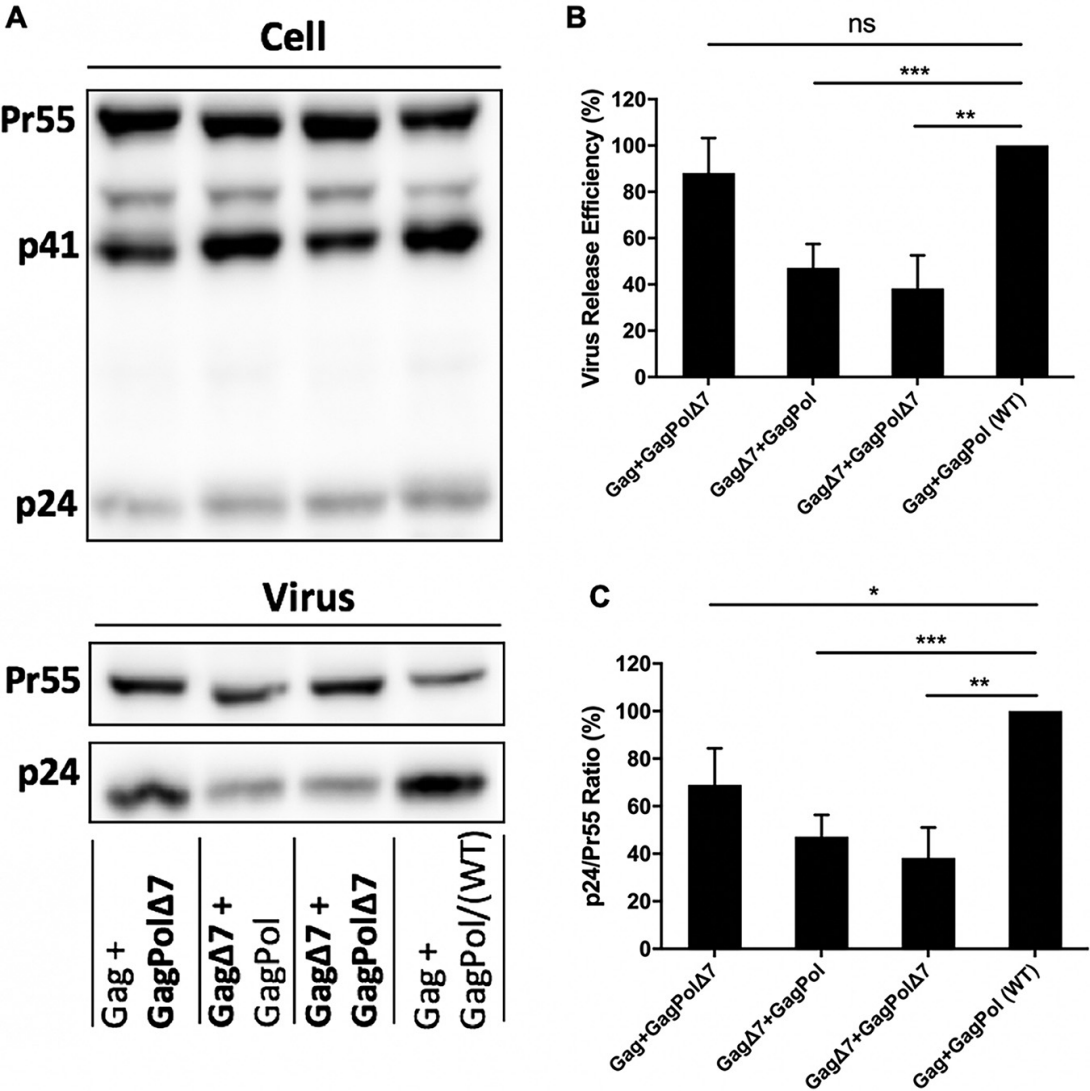
The role of the seven-amino-acid deletion in p6^Gag^ and the overlapping deletion in p6*. Detection of cell- and virion-associated proteins by WB analysis. **(**A) 293T cells were co-transfected with HIV-1 proviral clones that encode Gag and GagPol at a ratio of 15:1. Two days post-transfection, virus and cell lysates were harvested and measured by WB. Virus release efficiency (B) and Gag processing (C) were calculated as described in Figure 3. Virus production for WT was set as 100%. Standard deviation was obtained from more than three independent experiments; ns, not significant. *, *P* < 0.05, **, *P* < 0.01, and ***, *P* < 0.001.

The 7-aa PIDKELY deletion in p6^Gag^ also results in deletion of amino acids DRQGTVS in p6* due to shift of the open reading frame (Fig. 2). To determine the effects of the deletion mutation in p6^Gag^ and p6* in virus release and Gag processing, we constructed several HIV-1 pNL4-3/KFS clones expressing p6^Gag^ with the 7-aa deletion (GagΔ7) and p6* with the 7-aa deletion (GagPolΔ7). The GagPol construct expresses GagPol but does not express Gag, as the result of a 1-nucleotide insertion in the frameshift region that places gag and pol in the same ORF (14). The Gag- and GagPol-expressing plasmids were co-transfected into 293T cells at a ratio of 15:1 to generate viral particles with similar efficiency of virus release and maturation as normal HIV-1 particles (Fig. S3). We found that the deletion mutation in p6* did not significantly inhibit virus particle production or Gag processing (Fig. 5). In contrast, the deletion in p6^Gag^ resulted in a decrease in virus release and Gag processing efficiency to ~47% of the WT level (Fig. 5B, C). Furthermore, the deletions in p6^Gag^ and p6* did not affect the incorporation and processing of GagPol protein (Fig. S4 in the supplemental material). These results indicate that the 7-aa deletion in p6^Gag^, but not the deletion in the p6*, impairs virus release and Gag processing.

### The defects of HIV-1 CRF07_BC were caused by disrupting the binding of p6Gag and Alix.

**FIG 6:**
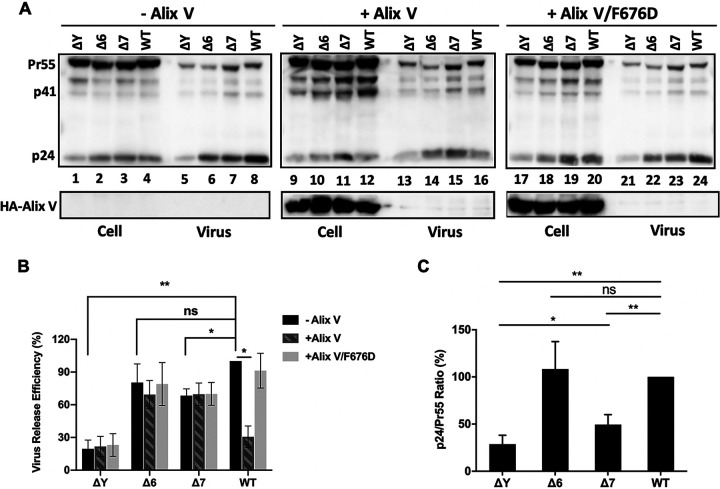
The p6 mutants are resistant to inhibition by Alix V overexpression. (A) 293T cells were co-transfected with HIV-1 proviral DNA encoding WT Gag or the deletion of the 36^th^ residue (ΔY), the 6-aa deletion (Δ6), or Δ7 mutant in p6^Gag^, together with control plasmid DNA (pcGNM2-Alix) or HA-Alix V or HA-Alix V/F676D expressing vector. At 24h post-transfection, cell-associated and virus-associated proteins were measured by WB. Pr55Gag, p41, p24 and HA-Alix V are indicated. The efficiency of virus release (B) and Gag processing (C) was calculated as described in Figure 3. The levels of WT virus produced in the absence of Alix V (-Alix) was set as 100%. Error bars show SD from four experiments. ns, not significant. *, *P* < 0.05, **, *P* < 0.01.

Previous studies reported that the Alix binding domain _36_YPXnL_41_ in p6^Gag^ promotes virus release through the interaction between HIV-1 p6^Gag^ and host protein Alix(15). The central, so-called “V” domain of Alix is responsible for binding the _36_YPXnL_41_ in p6^Gag^ (15–17). As a result, overexpression of the Gag-binding V domain of Alix (Alix V) potently disrupts particle budding by binding directly to HIV-1 Gag (16, 18, 19). As expected, virus release of HIV-1 WT was significantly inhibited by overexpressing Alix V in 293T cells. The Alix V/F676D protein, an Alix V variant that contains a Phe-to-Asp substitution in Alix residue 676 that abrogates p6 binding (16), did not inhibit particle release (Fig. 6A and B). Notably, overexpression of Alix V did not affect virus release of the three p6^Gag^ mutants analyzed: ΔY, Δ6, or Δ7 (Fig. 6A and B), indicating that these mutations prevent the interaction between p6^Gag^ and Alix. These data suggest that the deletion mutations in p6^Gag^ inhibit virus release by disrupting the binding of p6^Gag^ and Alix.

## DISCUSSION

HIV-1 CRF07_BC originated from co-infection or superinfection of HIV-1 subtype B’ and C (4, 20, 21). Now it is becoming increasingly prevalent and is one of the most common CRFs in China especially in HIV-infected MSM (22–25). Previous studies identified some new features of CRF07_BC including low viral loads, slow disease progression and potential longer life-time for CRF07_BC-infected subjects. In this study, we found that compared with CRF01_AE and subtype B, CRF07_BC and the recently emerged CRF55_01B have stronger transmission capability based on the growth rate values of HIV-1 transmission clusters. The new finding about the enhanced transmissibility of CRF07_BC and CRF55_01B is consistent with the increased prevalence of these two HIV-1 strains.

It has been clearly demonstrated that higher HIV-1 viremia leads to more efficient virus transmission among individuals. However, lower pathogenicity that is indirectly resulted from lower viremia or caused by the infection of HIV-1 with impaired replication may indeed contribute to virus spread in the population. Therefore, two possibilities may explain the rapid expansion of CRF07_BC. One is the enhanced transmission capability of CRF07_BC compared with HIV-1 subtype B or CRF01_AE. Another one is that CRF07_BC virus that is replication impaired in virus replication and infectivity may decrease viral loads, slow disease progression, and potentially extend lifetime and enlarge the pool of CRF07_BC-infected subjects. More CRF07_BC-infected individuals will potentially increase the risk of virus circulation. This may cause the fast spread of CRF07_BC in China.

In fact, Lin et al. has reported the signature mutation of 7-aa deletion at p6^Gag^ can affect virus production and decrease virus replication (11). In this study, we further characterized the biological significance of the 7-aa deletion mutant, and investigated another unique mutation in p6^Gag^, i.e. PTAPPE insertion, and the role of the double mutations in virus replication and infectivity. Our results showed that the 7-aa deletion, not the PTAPPE insertion, moderately reduce virus release and Gag processing, and resulted in defects in HIV-1 infectivity and replication in both T-cell lines and PBMCs by disrupting the interaction between p6^Gag^ and the host Alix protein. Our study provides further evidence for the important role of the interaction between HIV-1 p6^Gag^ protein and Alix binding domain in regulating virus release, Gag processing and replication, which in turn may explain at the molecular level the slower disease progression observed in individuals infected with CRF07_BC with the unique 7-aa deletion mutation in p6^Gag^.

The primary role of HIV-1 p6^Gag^ is to regulate virus budding by recruiting the ESCRT apparatus through the interaction between the late (L) domains of p6^Gag^ and host factors Tsg101 and Alix to catalyze the membrane fission reaction that allows the virus to pinch off from the plasma membrane (26). The two L domains in HIV-1 p6^Gag^ are the Tsg101-binding site, _7_PTAP_10_, and the Alix-binding site, _36_YPLASL_41_. Insertions into, or duplication of, the PTAP motif could enhance the interaction between Gag and Tsg101. Sharma et al. reported that 94.9% of p6^Gag^ sequences of HIV-1 subtype C carry the duplication of the PTAP motif. They confirmed that duplication of the PTAP motif enhances virus replication fitness, but not virus release of HIV-1, by binding the Tsg101 protein with a higher affinity (27). PTAP duplication is usually observed in HIV-1 strains with drug resistance mutations (28–31). Martins et al. demonstrated that PTAP duplication enhances virus infectivity by increasing PR-mediated processing between NC and p6 in the presence of PR mutations and PR inhibitors (PIs) (14). Martins et al. also found that the PTAP duplication did not increase virus release or the incorporation of *pol* products in virions. Tamiya et al. demonstrated that the PTAP insertion near Gag cleavage sites could restore the replication competence of multi-PI-resistant HIV-1 variants by enhancing the otherwise compromised enzymatic activity of mutant PR (31). In this study, we confirmed that the PTAP duplication alone does not affect virus release, infectivity, or replication. These results are consistent with those obtained in previous studies and indicate that the major role of PTAP duplication may be to restore the replication capability in the presence of drug resistance mutations.

Interestingly, we recently reported that HIV-1 CRF07_BC Gag with 7-aa deletion could induce stronger CD4+ and CD8+ T-cell immune responses, suggesting that CRF07_BC virus with 7-aa deletion in p6^Gag^ might enhance its cellular immunity and impact its pathogenicity (32). It has been reported that HIV-1 infection could activate immune responses and the continuous immune activation results in rapid differentiation of a subset of CD4+ and CD8+ T cells and activation-induced cell death (33–35), which may cause elimination of HIV-1 reservoir. The persisting slow-replicating viruses associated with a low virus load may be adapted to withstand cellular immunity (36). Patients infected the impaired HIV-1 may maintain slow disease progression but relatively stable size of HIV-1 reservoirs (37). Although the unique mutations in the HIV-1 p6^Gag^ were adapted from CRF07_BC, in our study, all the characterizations were conducted using the pNL4-3 HIV-1 molecular clone, which is based on the subtype B rather than CRF07_BC. The sequence analysis indicates that CRF07_BC is the combination of HIV-1 subtype B and C while the p6^Gag^ of CRF07_BC is adapted from HIV-1 subtype B (4, 38). Ideally, we should utilize the full clone of CRF07_BC with/without the mutations to investigate their virological features. We would like to emphasize that one purpose of this study is to evaluate the role of the two unique mutations in the p6^Gag^ protein in regulating virus replication, rather than comprehensively characterizing CRF07_BC. Therefore, we must interpret with caution the findings in our study.

In summary, in this study we confirmed the enhanced transmissibility of HIV-1 CRF07_BC and characterized two unique patient-derived mutations, a PTAPPE insertion and a PIDKELY deletion, in the p6 domain of HIV-1 Gag. Our results demonstrated defects induced by the 7-aa deletion mutation and the double mutations in p6^Gag^ on virus release, Gag processing, infectivity, and replication kinetics, at least in part, due to disruption of the p6-Alix interaction. The 7-aa deletion and 6-aa insertion in the p6 late domain can also change the interaction with Alix protein. Our findings help define the molecular mechanism regarding the association between the unique 7-aa deletion mutation and the interaction of the two unique mutations in p6^Gag^ and the slower disease progression observed in subjects infected with the Δ7 mutant CRF07_BC. The slower disease progression caused by CRF07_BC and the prolonged survival of infected persons may increase the total number of HIV-1 infected persons in the population and increase the risk of HIV-1 epidemic. Our results indicate that HIV-1 CRF07_BC-infected subjects require greater attention and effective intervention.

## MATERIALS AND METHODS

### Transmission network construction.

A total of 3079 HIV-1 pol sequences (HXB2 position 2253–3821) were collected from 2008 to 2015 in Guangzhou and used for transmission network analysis. These sequences were obtained from 3684 HIV-1 positive individuals who were diagnosed with HIV-1 infection through heterosexual transmission (HET), MSM, or intravenous drug users (IDUs). The detailed information about these HIV-1 sequences had been described in our previous study (39) and were available under the following GenBank accession numbers: MN424584–427369. The Tamura-Nei 93 nucleotide substitution model was used to calculate pairwise genetic distance. A putative link was inferred by HIV-TRACE with a pairwise genetic distance of 1.5% (40), which corresponds to a maximum of approximate 7-8 years of viral evolution. Multiple links were then resolved into transmission clusters.

### Determination of cluster growth.

To determine the growth and expansion speed of HIV-1 transmission clusters, we adapted the growth rate of transmission clusters, which is measured by dividing the number of newly diagnosed HIV-1 cases linked in a transmission cluster in the previous 12 months by the square root of the cluster size (41). For the unclustered HIV-1 sequences, the growth rate is 0. HIV-1 transmission clusters were classified into three categories according to the distribution of the growth rate values for all the clusters: high growth clusters with top 25% of the growth values, medium (25~50%), and low (≥50%), respectively.

### p6^Gag^ mutant construction.

The molecular clone pNL4-3 and the envelope (env)(-) derivative pNL4-3/KFS (42) of HIV-1 were obtained through the NIH AIDS Reagent Program. Different mutations in p6Gag were introduced into pNL4-3 by polymerase chain reaction (PCR)-based mutagenesis to generate the following three mutants: PTAPPE insertion (insPTAP), 7-aa (_30_PIDKELY_36_) deletion (Δ7), and the double mutant (PΔ7) containing both insPTAP and Δ7 (Fig. 2A and B). A PTAP motif deletion mutant (_7_PTAP_10_) was used as a control for measuring defective virus release (Fig. 2B). Three additional mutations were introduced into pNL4-3/KFS: ΔY, in which Y36 of p6^Gag^ was deleted; and Δ6 and Δ7, in which 30PIDKEL35 or 30PIDKELY36 of p6^Gag^ were deleted, respectively. An additional set of mutants was constructed to express Gag and GagPol polyproteins with the Δ7 mutation in p6^Gag^ or p6*, respectively, by using the plasmids pR7WT-HA and pR7insFS, which express the Gag and GagPol polyproteins, respectively (14). The Δ7 mutation in p6^Gag^ and p6* was then introduced into pNL4-3/KFS. All the constructs were characterized by restriction digestion analysis and DNA sequencing. The plasmids that express the V domain of Alix (residues 364-716, Alix V) and the Alix V derivative containing the F676D mutation (Alix V/F676D) have been described (16, 18).

### Cell culture and transfection.

293T and TZM-bl cells were cultured in Dulbecco’s modified Eagle’s medium (DMEM) supplemented with 10% (vol/vol) fetal bovine serum (FBS), 100 U/ml penicillin, 100 g/mL streptomycin, and 2 mM L-glutamine (Gibco). TZM-bl is a HeLa-derived indicator cell line that expresses luciferase following HIV-1 infection (43). Sup-T1, MT-4 T-cells, and PBMCs were cultured in RPMI 1640 medium supplemented with 10% (vol/vol) FBS, 100 U/mL penicillin, 100 g/ml streptomycin, and 2 mM L-glutamine. PBMCs obtained from anonymous, de-identified NIH blood donors were activated in RPMI 1640 medium supplemented with interleukin-2 and phytohemagglutinin (PHA) prior to HIV-1 infection. Adherent cells were transfected with plasmid DNA using Lipofectamine 2000 (Invitrogen Corp. Carlsbad, CA) according to the manufacturer’s recommendations. Cells and viruses were harvested 24 h post-transfection and used for further analysis.

### Virus release and maturation.

293T cells were prepared at 0.5 million per well in a 6-well plate one day before transfection and were transfected with 2.5 μg of WT or mutant pNL4-3 molecular clones using Lipofectamine 2000 transfection reagent. At 24-h post-transfection, virus-containing supernatants were collected and were pelleted by ultracentrifugation. Both cell and virus pellets were lysed in a buffer containing 50 mM Tris-HCl (pH 7.4), 150 mM NaCl, 1 mM EDTA, 0.5% Triton X-100, and protease inhibitor cocktail (Roche Life Sciences, Basel, Switzerland). After denaturation, proteins were subjected to SDS-PAGE, transferred to a polyvinylidene fluoride (PVDF) membrane, and incubated with HIV-1 immunoglobulin (HIV-Ig) obtained from the NIH AIDS Reagent Program. The membrane was then incubated with horseradish peroxidase (HRP)-conjugated secondary antibodies, and the chemiluminescence signal was detected by using Western Pico substrate (Thermo Scientific) or Western Femto substrate (Fdbio Science). Quantification of the protein band intensity was performed using ImageLab software (Bio-Rad). Virus release efficiency was calculated as the amount of virion-associated p24 (CA) as a fraction of the total amount of Gag including cell-associated-p24 and Pr55Gag plus virion-associated p24, or the RT activity of culture supernatants relative to WT level (44, 45). Virus maturation was measured by Gag processing and expressed as a ratio of virion-associated p24 over Pr55Gag levels as described previously (9, 46).

### Virus replication and infectivity.

Multi-cycle replication assays were performed using the Sup-T1 or MT-4 T-cell lines, and PBMCs. For Sup-T1 and MT-4 cells, 5 μg of plasmid DNA were transfected into 5 million cells using DEAE-dextran reagent (44, 47). PBMCs from multiple donors were infected with virus supernatants generated by transfecting 293T cells. Virus inputs were normalized by RT activity. Cells were infected by inoculation of HIV-1 viruses for 2 hours at 37°C. Virus replication was monitored by measuring RT activity or p24 concentration (48). Virus infectivity was monitored by measuring luciferase activity in TZM-bl cells infected with HIV-1 virus supernatants from 293T cells (14).

### Statistical Analysis.

Demographic characteristics of HIV-infected subjects were analyzed using Kruskal-Wallis test, Fisher’s exact test and chi-square test while cluster growth was compared with Kruskal-Wallis test followed by post-hoc test with Bonferroni procedure. Logistic regressions were conducted to determine the factors associated with the cluster growth and the speed of transmission. Gender, age, marital status, education level, occupation, infection routes and HIV-1 subtypes were included as predictor variables for cluster growth. Age was included in the analysis as a categorical variable. The factors were removed from the multivariate model if *P* > 0.1, using stepwise progress. The logistic regressions for cluster growth rate were performed with cutoffs of the 75th and 50th percentiles, respectively. To strengthen the robustness, sensitivity analysis was performed for the MSM population. Unpaired t tests were performed and two-tailed *, *P* < 0.05, **, *P* < 0.01, and ***, *P* < 0.001 were considered statistically significant. All the analysis was conducted in IBM SPSS Statistic 25.
