# Supplementary material for: Enhanced Transmissibility and Decreased Virulence of HIV-1 CRF07_BC May Explain Its Rapid Expansion in China
Source: Microbiol Spectr. 2022 Jun 21;10(4):e00146-22. doi: 10.1128/spectrum.00146-22 (PMC9431131; doi:10.1128/spectrum.00146-22)
Supplement: Supplemental file 1 — Fig. S1-S4; Tables S1-S3. Download spectrum.00146-22-s0001.pdf, PDF file, 0.3 MB [file spectrum.00146-22-s0001.pdf]

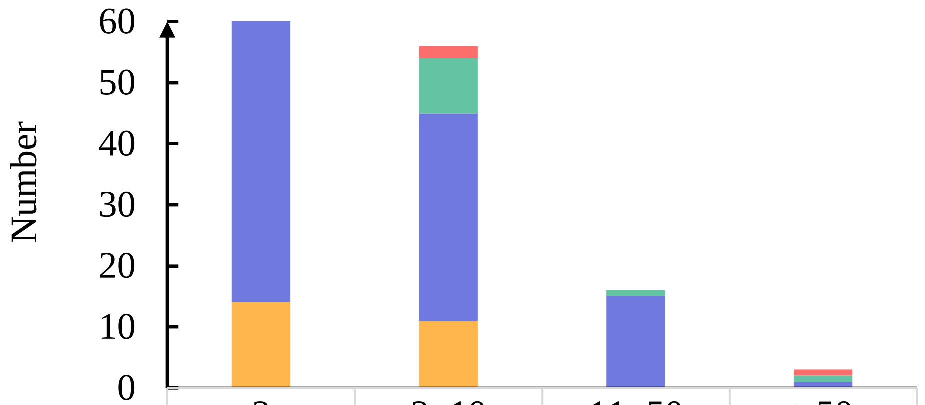

	2	3~10	11~50	>50
CRF55_01B	5	2	0	1
CRF07_BC	28	9	1	1
CRF01_AE	50	34	15	1
B	14	11	0	0

Cluster size

Figure S1 The distribution of cluster size in Guangzhou, China.

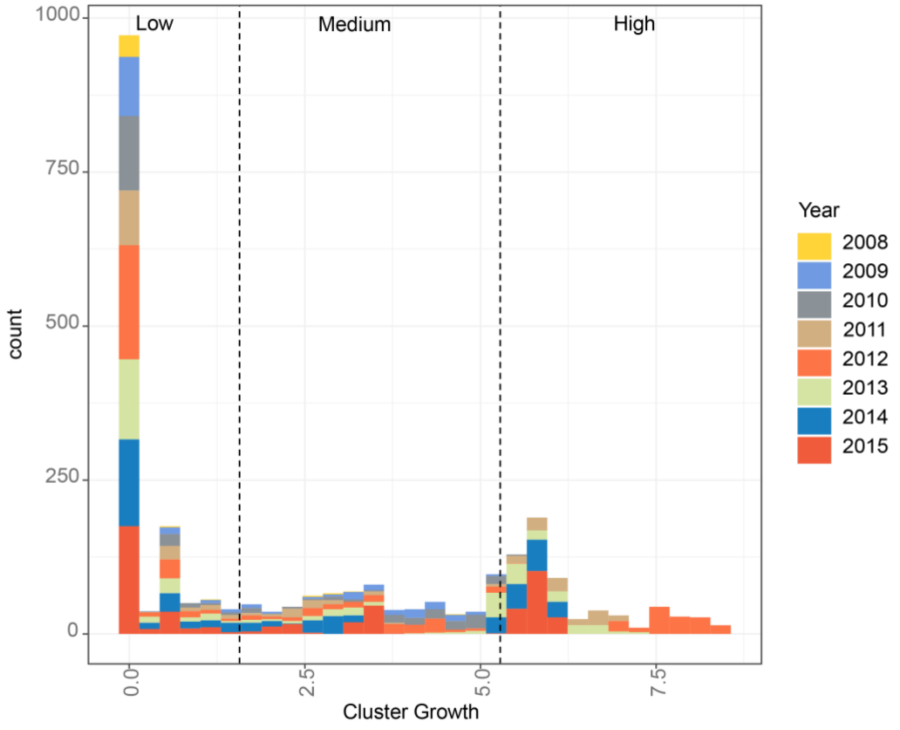

Figure S2 The distribution of cluster growth of each HIV-infected individual diagnosed during 2009 to 2015 in Guangzhou, China

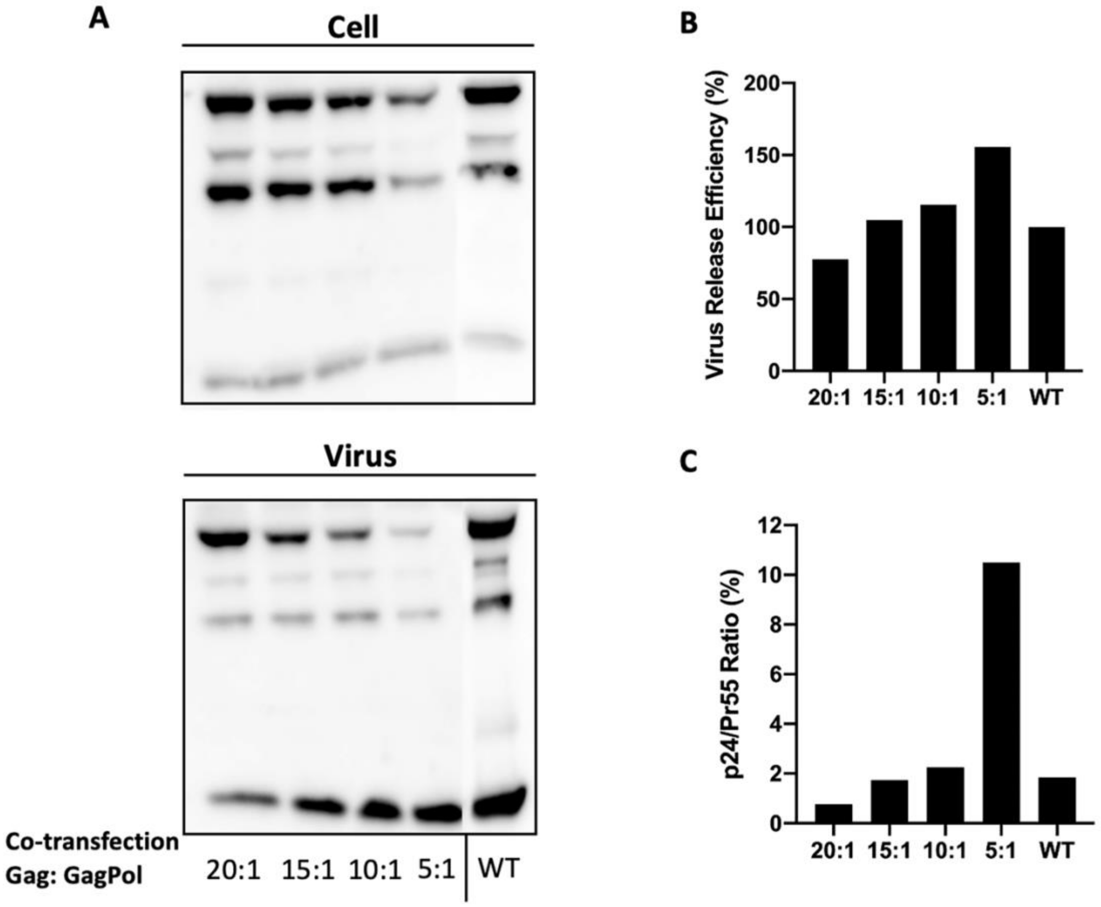

Figure S3 Virus release and Gag processing generated at different co-transfection ratios of Gag- and GagPol-expressing plasmids. **(A)** 293T cells were transfected with HIV-1 pNL4-3 clone (WT) or co-transfected with HIV-1 proviral clones that encode Gag and GagPol at a ratio of 20:1, 15:1, 10:1 or 5:1. Two days post-transfection, virus and cell lysates were harvested and measured by WB. Virus release efficiency **(B)** and Gag processing **(C)** were analyzed as described in Figure 3. The virus release of WT was set as 100%. Data was obtained from one experiment.

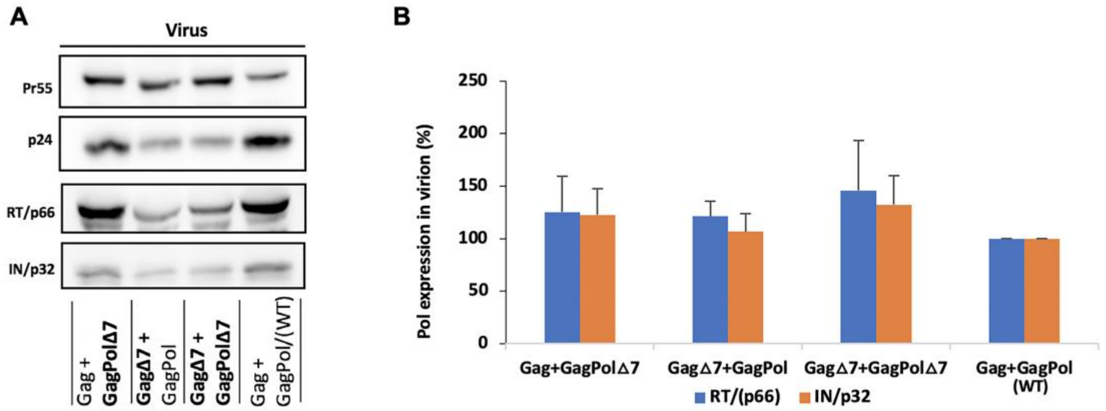

Figure S4 The role of the seven-amino-acid deletion in $p6^{Gag}$ and the overlapping deletion $p6^{*}$ in packaging of Pol products. (A) A gel run of Western blotting. 293T cells were co-transfected with HIV-1 proviral clones that encode Gag and GagPol at a ratio of 15:1. Two days post-transfection, virus and cell lysates were harvested and subjected to Western blotting detecting Gag and Pol products. Panel A is part of Figure 5 in which did not shown the Pol products. (B) Quantitative analysis of Pol products in Western blotting. The level of RT(p66) and IN(p32) in virion were quantified and normalized to the level of virion-associated Gag (Pr55+p24), respectively. The WT, generated from co-transfection of WT Gag plus WT GagPol, was set equal to 100%. Standard deviation was obtained from more than three independent experiments.

Table S1 Factors associated with the growth index of HIV-1 transmission clusters among MSM using overall models.

Characteristics	cluster growth ≥ 1.57		cluster growth ≥ 5.28	
	OR (95%CI)	*P*	OR (95%CI)	*P*
Age group				
below 20	1.080 (0.617, 1.892)	0.788	1.323 (0.675, 2.591)	0.323
21~30	ref		ref	
31~40	0.896 (0.648, 1.240)	0.509	0.956 (0.655, 1.394)	0.779
41~	0.573 (0.353, 0.929)	0.024	0.747 (0.445, 1.256)	0.186
Marital status				
Unmarried	ref		ref	
Married	1.323 (0.885, 1.978)	0.172	1.239 (0.793, 1.937)	0.199
Divorced	1.764 (0.920, 3.383)	0.087	1.537 (0.756, 3.126)	0.251
Not disclosed	1.259 (0.569, 2.786)	0.570	0.612 (0.210, 1.780)	0.540
Education level				
Primary or below	ref		ref	
Junior or High	1.140 (0.530, 2.450)	0.737	1.020 (0.550, 1.893)	0.728
College or Others	1.096 (0.498, 2.412)	0.820	1.056 (0.539, 2.068)	0.975
Not disclosed	1.417 (0.432, 4.652)	0.566	0.228 (0.062, 0.842)	0.612
Occupation				
Unskilled work	ref		ref	
Skilled/Professional work	1.945 (1.225, 3.089)	0.005	1.200 (0.688, 2.092)	0.157
Students	1.217 (0.702, 2.109)	0.484	1.018 (0.513, 2.019)	0.436
Unemployment	0.852 (0.517, 1.406)	0.532	1.014 (0.629, 1.634)	0.141
Not disclosed	1.060 (0.770, 1.458)	0.721	1.225 (0.828, 1.813)	0.936
Genotypes				
B	0.068 (0.021, 0.216)	<0.001	-	-

CRF01_AE	ref		-	-
CRF07_BC	25.195 (18.863, 33.654)	<0.001	ref	
CRF55_01B	26.119 (17.222, 39.611)	<0.001	0.014 (0.008, 0.024)	<0.001

Table S2 Factors associated with the growth index of HIV-1 transmission clusters among MSM using stepwise models.

Characteristics	cluster growth ≥ 1.57		cluster growth ≥ 5.28	
	OR (95%CI)	*P*	OR (95%CI)	*P*
Education level				
Primary or below	-	-	ref	
Junior or High	-	-	0.578 (0.205, 1.630)	0.300
College or Others	-	-	0.526 (0.186, 1.481)	0.224
Not disclosed	-	-	0.078 (0.018, 0.338)	0.001
Occupation				
Unskilled work	ref		-	-
Skilled/Professional work	1.884 (1.205, 2.944)	0.005	-	-
Students	1.220 (0.736, 2.023)	0.441	-	-
Unemployment	0.846 (0.512, 1.396)	0.512	-	-
Not disclosed	1.026 (0.755, 1.395)	0.870	-	-
Genotypes				
B	0.067 (0.021, 0.214)	<0.001	-	-
CRF01_AE	ref		-	-
CRF07_BC	24.637 (18.493, 32.822)	<0.001	ref	
CRF55_01B	24.965 (16.621, 37.497)	<0.001	0.008 (0.004, 0.017)	<0.001

Table S3 Comparison of HIV-1 Transmission Growth Index according to HIV-1 Genotypes among MSM

HIV-1 Genotype	HIV-1 Transmission Growth Index			*H*, *P* value*
	No. analyzed (%)	Median (quartile)	Mean rank	
Subtype B	123 (6.38)	0.00 (0.00, 0.50)	351.07	
CRF01_AE	714 (37.05)	0.58 (0.00, 1.70)	537.20	$H = 1164.92$, $P <$ 0.01
CRF07_BC	782 (40.58)	5.80 (5.34, 6.17)	1441.81	
CRF55_01B	308 (15.98)	3.38 (2.83, 3.85)	985.03	
Total	1927 (100.00)	3.02 (0.00, 5.62)	-	

Cluster growth was measured by calculating the number of newly diagnosed cases linked in a cluster in the previous 12 months divided by the square root of the cluster size. * Kruskal-Wallis test. (Post-hoc test by Bonferroni)
